# Surgery

**Published:** 1859-11

**Authors:** 


### Bi-Monthly Periscope.

### SURGERY.

1. Excision of the Testis for Malignant Disease.—[In the Medical Times and Gazette of the 17th of September, we find a tabular statement of thirty-six cases of malignant disease of the testis, in all of which, with one exception, the gland was removed. As the table is too long for insertion, we can only add the comments upon these cases.]

*Forms of Cancer to which the Testis is liable.*—Of the above thirty-six cases it would appear that in almost the whole the form of cancer was the soft form of medullary. In several the structure of the diseased mass was firmer than that of a typical medullary growth, but in none did it approach that of true scirrhus. Modern histological investigations have indeed tended to show that true scirrhus, such as that so commonly seen in the mammary gland, for instance, is never met with in the testis. The supposed instances of it described by Sir Astley Cooper and other writers have proved, on microscopic examination, to be not cancer, but cartilage. It is quite possible also that examples of syphilitic sarcocele, with an unusual degree of induration, may occasionally have been regarded as scirrhus.

In Case 9 the original disease of the testis itself was simply cartilaginous, and the subsequent deposits in internal organs, which were very numerous, were of the same material. As to the malignancy of the disease in this case, there can, however, be no doubt, as the man died of its development in the lungs within a short period of the operation.

In Case 5 a "fibroid" tumor had been excised three months before from the scrotum, and the recurred one, which had involved the testis, if it did not originate in it, was of the same nature. This case probably stands on the border ground between true cancers and recurrent fibroids, and there is room for question as to the propriety of its being included in this series.

Cases 8, 11, and 18 are examples of the coexistence of medullary cancer, with well-characterized cystic development.

*Age most liable to Malignant Disease of the Testis.*—It would appear that all periods of life, excepting the senile, and including even those of infancy, are liable to the occurrence of malignant disease of the testicle. In the youngest patient in our list it began at the age of one year and eight months, while the oldest was 59. Taking the decennial periods, we have three instances of the disease occurring between the first and tenth years, one between 10 and 20, eight between 20 and 30, thirteen between 30 and 40, six between 40 and 50, and five between 50 and 60. The age stated in each case is that at which the patient came under surgical treatment for his disease, and the age at which the latter had first commenced was most probably about a year younger. Bearing this fact in mind, we can have no hesitation in accepting the conclusion, that the first half of adult life (from 20 to 40) is the period in which the testis is most liable to be attacked by medullary cancer.

The instances in which the disease began in early boyhood have a peculiar interest. It is an important fact that the testis should be so liable as it is to the attacks of disease (benign as well as malignant) at periods considerably prior to its full development, and to the assumption of functional activity. Parallel instances of disease in the ovaries or mammary glands of preadolescent girls are undoubtedly far more rare. The converse comparison holds good as it regards the age at which the function of the gland in question has either ceased or is greatly on the wane. We have in the present series no example of cancer of the testis later than the age of fifty-nine, while but a very small proportion of the whole are met with so late as the fifth decade. In females, as is well known, diseases of the breasts and ovaries are still very frequent.

*Usual Rate of Progress of Cancer of the Testis.*—Malignant disease of the testicle has often been taken, and very properly so, as a typical example of cancer. It is acute in its onset, and very rapid in all its stages of progress. The facts recorded in the above series fully bear out these statements, and the inference is of great importance in its practical aspects.

In Case 21, the only one in which the disease ran its course without surgical interference, the patient was dead within four months of his having first noticed enlargement of the gland, at which time he was in what he considered robust health. The duration of the disease in Mr. Kidd's case, which was probably a similar one, was exactly the same.

In Cases 16, 36, 20, and 17, although an operation was performed, and in several of which it in all probability prolonged life, the whole duration from first to last was only six, nine, twelve, and twenty-one months respectively. In Cases 1, 3, and 27, which ended fatally within a week or two of the operation, deposits of cancer were found in the lumbar glands, which must have induced death within short periods, even if the patients had recovered from the immediate effects of the operation. The average previous duration of the disease in these cases had been only eighteen months. In two or three instances in which durations of unusual lengths are assigned to the disease, it is not improbable that the testis during the earlier portions of the time had not been affected by cancer, but by some inflammatory enlargement, on which at a subsequent period cancer had become ingrafted. It is not at all unusual for cancer to attack organs damaged by previous chronic inflammations; and the fact that in the cases adverted to a recent rapid increase in size had been observed, is in favor of our supposition.

*Treatment; immediate Results of the Operation of Excision.*—Although not a few and very different schemes of constitutional treatment have been proposed for the management of the more chronic cancers, none have, we believe, been suggested as likely to retard the progress of malignant disease of the testis. In this instance, the question as to treatment is simply, Shall extirpation be performed or not? the alternative being certain and speedy death. As might be expected, therefore, under such circumstances, the propriety of extirpating a testis affected with malignant disease, provided the cord be healthy, is one of the rules in surgery about which there is no dispute. The operation *per se* is one of little or no danger if done early, while the gland is not enlarged beyond a certain size, while the cord is healthy, and before the lumbar glands have been affected. In nearly all the cases in our series in which death followed as a consequence of the operation, large tumors were found in the abdomen, which had been unsuspected previously, but which had no doubt exercised a most prejudicial influence on the patient's chance of recovery. That excision of the testis in itself is not an operation attended by any material risk, is proved by the facts that of the above series 26 out of 36 recovered from it, while of the cases to follow this report, in which the testis was removed for diseases of non-malignant nature, the proportion of recoveries was 26 to 1 death.

In determining which cases are best suited for operation, and those in which for the credit of surgery it will be well to decline using the knife, regard should be had to the size and duration of the tumor, to the condition of the cord, to the patient's general health, and to the indications of disease of the lumbar glands. If there be good reason to think that the lumbar glands are already enlarged, or if the cord be positively enlarged at a point above where it could be divided, an operation could only end in disappointment. Too much weight in either direction must not be allowed to attach to the state of the patient's health. In not a few instances, owing to its rapid rate of progress, the disease has advanced beyond hope of benefit by surgery, before the patient's aspect has assumed any very decided indications of malignant cachexia. In many of the cases in our list the statement is, that "the man was in good general health." On the other hand, it is quite possible that a patient might appear very cachectic from accidental causes, while still his lumbar glands were free from disease. The large size of the tumor, although "equaling that of a cocoa-nut," would not, of course, in itself deter any surgeon from attempting its removal, other circumstances being favorable. It must be granted, however, that it renders the prognosis much more serious.

*Ultimate Result of Cases Operated upon.*—As just stated, five out of the thirty-five cases ended in death within a week or two of the operation, and either as its immediate or indirect consequence. In five others (Cases 7, 28, 30, 10, 36,) the patients did not, while under observation, regain their health, and in four of these the disease already appeared to be recurring, *i.e.* within a month or two of the operation. In Cases 17, 20, and 30, although the patients regained good health after the operation, yet the disease recurred subsequently in internal organs, and death took place at the respective periods of three and ten months afterwards. In Case 16 the patient died within six weeks of the operation; not, however, from its effects, but from recurred and rapidly advancing disease. In Case 6 the patient was known to be in good health, but with suspicious induration of the inguinal lymphatics, six months subsequent to his discharge from the hospital. These cases deducted, we have remaining twenty in which, beyond the facts that the patients left the hospital well within short periods of the operation, we know nothing. Not a few of these men have, in all probability, since died of internal cancer; while there is a fair foundation for the belief that others are still in good health.

Mr. Cock has mentioned to us the case of a man whose testis he removed for medullary cancer, and who remained under observation and in good health for six years afterwards, being at last lost sight of in consequence of his emigration to Australia. This fact is the most important in relation to this subject that has come within our knowledge in the practice of the London Hospital. Whole histories of cases of malignant disease of the testis are much wanted in the literature of clinical surgery.—*Med. Times and Gazette*, Sept. 17, 1859.

2. ON THE DIAGNOSIS OF THE SACRO-ILIAC DISEASE. By *Mr. Erichsen*, Professor of Surgery in University College, London.—Mr. Erichsen gives the name of *sacro-iliac disease* to disease of the articulation between the sacrum and the pelvis. The diagnosis, he tells us, is not always easy, and he points out five affections with which it may be confounded—namely, neuralgia of the hip, sciatica, spinal disease, coxalgia, and disease of the pelvic bones.

1st. *Neuralgia of the hip* in young females may readily enough be confounded with the earlier stages of sacro-iliac disease. But the widely-spread and superficial nature of the pain in the neuralgic affection, the coexistence of the hysterical temperament, the sex of the patient, and the absence of all limitation of morbid action to the neighborhood of the diseased articulation, render the true nature of the affection sufficiently clear. The obliquity of the pelvis, which occasionally occurs in neuralgia of the hip, and causes apparent elongation of the limb, is readily removed when the patient lies on her back; whereas, in sacro-iliac disease, position does not affect the displacement of the limb on the affected side.

2d. *Sciatica.*—In this affection, the age of the patient, usually more advanced than that of the subjects of sacro-iliac disease; the seat of the pain, below the articulation, and its extent down the back of the limb; with the absence of elongation, will enable the surgeon to effect the diagnosis.

3d. From *spinal disease*, the diagnosis is usually sufficiently easy, for although the situation of abscess resulting from caries of the vertebræ may in many cases be the same as that which is occupied by the collections of pus resulting from "sacro-iliac disease," yet in caries of the spine, in the vast majority of instances, excurvation of the vertebræ has become prominently marked by the time that the abscess has assumed so great a magnitude as to occupy the inferior lumbar or gluteal regions. In those rare cases in which, as an instance that was recently under my care, caries of the vertebræ, with consecutive abscess, takes place without any angular curvature, it will be found that the patient complains of tenderness, on the surgeon percussing the spine opposite the seat of disease; that the spinal column has lost its flexibility, moving stiffly and as a whole; that there is an absence of that elongation of the limb on the affected side, dependent on displacement of the wing of the pelvis, which is so early observable in sacro-iliac disease; and lastly, that examination of the sacro-iliac synchondrosis neither elicits pain, nor reveals swelling, or any of the other signs of disorganization of that articulation.

4th. *Coxalgia* is the affection that is most easily confounded with sacro-iliac disease; and that from which it is of most importance to make the diagnosis. It is especially from that variety of hip-disease that commences in the acetabulum, that primarily involves the pelvic bones, and only secondarily implicates the joint, that it is difficult to distinguish sacro-iliac disease; and the importance of effecting this diagnosis is great when we reflect that these cases of hip disease may now successfully be subjected to operative interference, while sacro-iliac disease does not admit of relief or removal by these means. Now, the diagnosis between coxalgia in all its forms and the disease we are at present considering may be effected by attention to the following circumstances:—

(1.) The seat of pain on pressure. In hip disease the patient suffers most severely when pressure is exercised deeply behind and above the trochanter, in the hollow behind that osseous prominence, or when the compression is exercised against the anterior part of the hip-joint through the pectineus muscle. In sacro-iliac disease little or no pain on pressure is experienced in these situations; but tenderness is elicited by pressure upon the sacrum and along the line of junction between the sacrum and ilium behind and altogether away from the hip.

(2.) The movements that occasion pain are different in the two diseases. In hip disease, abduction and rotation outward, or pressure of the head of the femur into the acetabulum, aggravate, to a greater or less degree, often to an unbearable extent, the sufferings of the patient. In sacro-iliac disease the thigh, may be moved in all directions, abducted or adducted, rotated, flexed, or extended, while the patient is lying on her back, without any increase of suffering, *provided the side of the pelvis be fixed by the surgeon.* Should this precaution not be taken, the movement impressed on the thigh will be communicated to the diseased articulation and will necessarily occasion suffering.

(3.) The signs connected with the alteration in the length of the limb differ in the two diseases. In hip disease there may be, and usually is, in the advanced stages, considerable shortening. This never occurs in sacro-iliac disease. In the earlier stages of coxalgia there may be, as there is throughout in sacro-iliac disease, elongation of the limb. But there is an important point connected with this. The elongation in hip disease is always appreciable by measuring from the anterior superior spine of the ilium to the inner ankle. In sacro-iliac disease, however, the measurement between these two points on the opposite sides of the body exactly corresponds, the seat of the elongation being situated still higher up.

(4.) The alteration of the level and of the prominence of the two anterior superior spines, in sacro-iliac disease, may be confounded with that arising from the obliquity of the pelvis usually occurring in the early stages of coxalgia. But here also the diagnosis may be effected by observing that the displacement of the bone in sacro-iliac disease is permanent, and is not influenced by position. The obliquity of the pelvis in hip disease, giving rise to apparent elongation of the limb, is dependent on a twist in the lumbar spine, which may be rectified by placing the patient on his back, and using a little manipulation. The alteration in the level of the two ilia, in sacro-iliac disease, is not modified by change of position, or by any movement that may be impressed upon the spine.

5th. *Disease of the pelvic bones* may of course occur independently of any affection of the sacro-iliac articulation, and when so occurring, it always commences at a distance from it, the crista ilii, the tuber ischii, or the acetabulum, being the usual seat of the disease. When occurring in the first of these two situations, the resulting abscess seldom attains a very large size, and is altogether above or below the synchondrosis, the outline of which can be felt clear and unobscured by swelling of any kind. When the abscesses are opened, the sinuses that result will lead directly down to the rough and carious bone, examination of which will leave no doubt as to the nature of the cases. In these cases, also, no change takes place in the length of the limb, or in the position of the side of the ilium.

When the acetabulum is primarily affected, the difficulty of diagnosis may be greater, in consequence of the large size and often infra-pelvic nature of the abscesses, and the coexistence of a certain amount of displacement or elongation of the limb. But here the same circumstances that enable the surgeon to effect a diagnosis in ordinary coxalgia—viz., the pain in movement influencing the hip-joint merely, and the increased length of limb, as determined on measuring from the anterior superior spines—will prevent his falling into error as to the true nature of this disease.—*Ranking's Abstract*, No. 29, 1859.

3. REPORT OF A CASE OF EXTIRPATION OF THE TONGUE. By *Alexander Fiddes F.R.C.S.E.*, Kingston, Jamaica.—The tongue, in common with other organs and tissues of the body, is liable to become affected by malignant deposits, producing a form of disease as ill to suffer and as surely fatal as any other affliction of humanity; for whether the morbid action appear on the surface of the organ as epithelial cancer, or as a circumscribed induration or tumor in its substance, or as a more general infiltration of the texture, little or no benefit is derivable from any kind of local application, or from any plan of constitutional treatment.

The highest surgical authorities agree as to the efficiency of therapeutic measures, in the diseases of the tongue that are truly cancerous; and they also concur regarding the uselessness of removing by the knife, or by ligature, the portion of the organ in which the disease may seem to be confined. Such operations are, indeed, described in several surgical works, as if they were justifiable and warrantable procedures, but they are probably never followed by a permanently beneficial result; on the contrary, they have been found rather to give an impetus to the morbid action, and to accelerate the fate of the patient.

Mr. Travers considered that carcinoma of the tongue admits only of palliative treatment, and never of operative interference, as in all the cases of partial removal with which he was acquainted, with the exception of one, the disease had returned within a short time; and Sir Benjamin Brodie, in a letter on this subject, speaks in language equally decided and despondent. He says: "There is no great difficulty in removing, either by the knife, or by ligature, any tumor from the tongue, except it be situated just at the back; but then I must tell you that I never saw any permanent good arise from it in any one instance." And in the fourth edition of the *Principles of Surgery*, Mr. Syme observes: "When the cancer is seated on the edge of the tongue, it may be safely cut out with the knife or scissors, after being grasped and forcibly stretched by a hook or hooked forceps. But the result of experience forbids almost any hope of effecting permanent relief by extirpating cancer even of this part, and of course, if possible, still less, when the disease is seated farther back."

It was, doubtless, from a conviction of the uselessness of any partial removal, and of every remedial measure hitherto employed, that Mr. Syme was induced, a short time ago, to propose the excision of the entire organ, and to put it in practice in two cases.* But the result in both instances was unsatisfactory; for the first patient died on the seventh day, the other on the fourth day, after the operation; and in consequence of these failures, this eminent surgeon has interdicted, with his high authority, any further attempts of a similar kind. But, probably, better success might have attended these trials if the patients had come under surgical treatment at an earlier period of the disease, when their constitution had suffered less, and were, in all likelihood, better able to withstand the vital disturbance occasioned by the operation. That extirpation of the tongue will ever be made generally available, or be often warrantable, in cases of malignant degeneration, it would be rash and presumptuous to maintain; but that such a procedure is not necessarily fatal, and that it will sometimes prove adequate to rid the patient of the disease, and perhaps prevent the return of it, is, I think, made evident by the history of the following case. At the time when the subject of it came under my care, I was not in possession of Mr. Syme's report of his cases; for if I had been aware of the conclusion at which he arrived in reference to this proceeding, I would not have been disposed to practice an operation so unfavorably viewed by this great master of modern surgery:—

Fanny M'Cartney, aged thirty-five, consulted me in March, 1858, as to the state of her tongue. There was a sore seated about the centre of the organ, a little to the right of the mesial line; its circumference was about that of a six-

---

* Vide Lancet, August 14, 1858.

pence, and its surface was covered by an ashy-colored viscid secretion; it lay upon and was surrounded by a circumscribed and rather irregular induration, the size of a small French olive, and nearly of that shape. Toward the edge of the tongue, on the same side, and a little farther back, there was a second induration, smaller than the other, and without any ulcerated breach over it. She stated that her attention had been first directed to the sore about six months before, and that she had made use of several applications to it without any beneficial effect. She affirmed that she had not been affected with any venereal complaint, and that she had never been subjected to a mercurial treatment; nor were there any appearances on the skin, or to the throat, to lead to the suspicion of her having ever suffered from either of these deleterious agencies. She considered her health as tolerably good, though her personal appearance was thin, and somewhat indicative of constitutional derangement. She had very little pain or uneasiness in the part, but complained of the tongue feeling rather heavy and benumbed. She was ordered a wash for the mouth, with some alterative medicine and generous diet; and she went to a distant part of the island, to attend to the occupation in which she was engaged. Although, at this time, the character of the sore seemed very suspicious, I did not venture a decided opinion regarding it, but requested the patient to return, in the event of its not improving or getting worse. She came back to me in the beginning of August, considerably fallen off in health, and the sore presenting still more unequivocal indications of a malignant disposition; the pain, in particular, which was absent at first, had now become troublesome; it was of a lancinating or shooting character, extending toward the ear, and in the direction of the angle of the jaw; but the glands of the neck were unaffected, and the tongue was in nowise fixed or adherent to the floor or sides of the mouth, nor was the posterior part of the organ, so far as could be ascertained by examination, implicated by the disease. Considering the case to be a favorable one for complete excision, I submitted the patient to the inspection of several of my professional brethren in the city, and she was also examined by Dr. Lawson, Inspector of Her Majesty's Army in the colony; and finding that their opinions were unanimous and confirmatory of my own, as to the malignant nature of the disease, I had no hesitation in advising the removal of it. To this the patient consented; and on the 2d of September I performed an operation, the steps of which were as follows:—

*Operation.*—The patient was seated in an arm-chair, and the body secured to it by a sheet passed around. Chloroform having been administered, one of the lower central incisor teeth was extracted, and the lip divided in the mesial line to the lower margin of the chin; here several small vessels spouted from the cut surfaces, requiring ligatures, and by the time they were all secured, the effect of the chloroform had passed away. It was therefore re-administered; and as soon as she was brought fully under its influence, the jaw was sawn through at its symphysis, and the two halves held asunder by two assistants. Then grasping the tongue firmly with a vulsellum, it was held and stretched forward and upward by a third assistant, while I introduced two fingers of the left hand beneath it, and dissected it away from the floor of the mouth by a strong, blunt-pointed and slightly curved scissors, leaning the instrument on the fingers as I proceeded, and feeling with them what way was made in the separation of the parts. The line of dissection was carried a little obliquely, so that I divided the left lingual artery before cutting the right; and as soon as these vessels were tied, the dissection of the organ was proceeded with, as far as its attachment to the hyoid bone. Then taking a straight, sharp-pointed bistoury, I pushed its point through the firm fibro-cellular texture, constituting the root or osseous connection of the organ, at the same time feeling for, and meeting the point of the instrument with the end of the left forefinger, which had been passed over the upper surface of the tongue, and placed within the concavity of the *os hyoides*, in front of the epiglottis. Through the perforation thus made, I then passed a straight, blunt-pointed knife, and keeping the blade closely applied to the bone, and guiding it with the left forefinger, I separated the parts completely from their osseous insertion, and finally, divided the two lateral folds of mucous membrane connecting the tongue to the palate. As soon as the detachment was effected, two small vessels, lying in close proximity to the hyoid bone, bled and required tying; but there was no further hemorrhage from any part of the mouth.

The extirpation having been thus accomplished, the two halves of the maxillary bone were put together, and retained in apposition by a fine silver wire passed between the teeth. The lip was closed by sutures, and a bandage applied to support the jaw. In the latter stage of the operation, the action of chloroform could not be maintained, yet the patient bore the whole procedure remarkably well, and evinced very little vital shock or depression at its close. After her removal to bed, ice was applied around the jaw, and small pieces of it were from time to time placed within the mouth. Late in the evening it seemed necessary to supply some nourishment; and with this view, a flexible catheter was passed along the floor of the nostril; but as soon as the end of the instrument dipped into the pharynx, a paroxysm of irritation and cough was excited, which brought on some bleeding from the mouth, and obliged me to withdraw the tube. The bleeding was controlled by the application of ice; but I made no further attempt to give nourishment in this manner, but allowed her to take it in her own way from the spout of a small teapot. From this time the patient progressed favorably, taking nourishment in sufficient quantity, and sleeping very well at night, with the assistance of an opiate occasionally, but generally without it.

*Subsequent history.*—The wound gave but little trouble; the incision in the lip healed by the first intention; the ligatures in the mouth came duly away; and the raw surfaces granulated and healed. The only treatment required was the application of a bandage to support the jaw, and a chlorine wash to cleanse the mouth. It was rather more than two months, however, before the bone was firmly consolidated by osseous union; and somewhat later, three small scales of bone successively exfoliated from the chin; but with the exception of that annoyance, and some degree of salivation, which was rather troublesome at first, no untoward circumstance occurred to retard convalescence. Fully seven months have now elapsed since the operation, and the mouth continues perfectly sound; while there has been a progressive improvement in the health, in respect to which, the patient's present state contrasts very favorably with her former condition. The power of deglutition is now so little impaired, that she can swallow solid articles of food nearly as well as fluids; the sense of taste is blunted, but not abolished; and the faculty of speech, though considerably weakened, is still sufficiently preserved to enable her to engage in conversation,—her voice, though low, being distinctly audible to persons near her.

Whether the beneficial result thus obtained is to continue as a permanent cure, or prove only a temporary respite, may be still questionable; but a similar uncertainty always attends operations for cancer on other parts of the body; and whether this complaint is to reappear, after excision. in any given case, must be always more or less problematical. In the present instance, the operation has been successful so far; and whether malignant action show itself at some future period, or remain permanently absent, I shall, in either case, be disposed to think that operative interference was not unjustifiable or improper. No doubt, in a majority of the cases in which malignant action manifests itself in the tongue, no operative interference can be admissible; nevertheless, in those instances where the disease has not invaded the surrounding parts, but is limited, circumscribed, and confined within the tongue itself, it is probable that the excision of the whole organ may eradicate the local affection, and afford the patient a fair chance of escape from a very painful and miserable death. But as the operation is not only severe to the patient, but likewise rather formidable to those engaging in its execution, it should not be undertaken except by a surgeon who is conversant with operative practice, and can command the services of an assistant on whose manual dexterity he can rely. But a good deal of the pain and shock may be averted by chloroform, and the hemorrhage need not be great,—the only arteries of consequence that come in the way being the two lingual trunks, and they may be readily seized and secured; for the section of the maxillary bone opens up the mouth thoroughly, affords free access to the throat, and enables the operator to command any bleeding that can possibly arise. It is important, however, to secure one lingual artery before dividing the other, and not to cut both at the same time, as a simultaneous bleeding from the two sources might rapidly fill the mouth, obscure the view of the wound, and cause embarrassment in the application of the ligatures; but this contingency may be certainly avoided by carrying the dissection on one side of the organ a little in advance of that of the other.—(*Edinburgh Med. Journal*, June, 1859.)

4. DIVISION OF THE POPLITEAL NERVE FOR NEURALGIA IN THE LEG. By *E. M. C. Hooker, Esq., M.R.C.S.*, Hadlow.—Jane B., aged twenty-five, has suffered for the last ten years from the most excruciating pain in the left leg. The pain has been attended with atrophy of the muscles of the limb and ulceration, said to be peculiar to disease of the sensitive nerves. To obtain relief from this most miserable condition, she has three times been an inmate of the Maidstone Infirmary, once of St. Thomas's Hospital, once of St. George's Hospital, once of St. Bartholomew's Hospital, twice of Margate Infirmary, and three times of King's College Hospital, where she has had the tibia gouged in all directions; still no relief. The constant pain and almost entire loss of sleep, at last began to tell seriously on her strength; death seemed to be looming at no great distance. Although amputation had been most earnestly requested by the patient, none of the eminent surgeons under whose care she had been, considered it advisable to accede to her desire.

The only method of obtaining relief seemed to me to be section of the popliteal nerve. After consulting with my partner, Mr. G. Vine, I determined to cut down on and divide the nerve as high as one could conveniently do so. Accordingly, on the 24th of June, the patient being under the influence of chloroform, I traced the edges of the ham-string muscles from below upward, till I reached the point at which they appear to join. I then made an incision in a slightly oblique direction from without inward, carefully cutting until I reached the edge of the biceps; then the director was freely used, and in a few seconds the nerve was thoroughly exposed and cut. The edges of the wound were then brought together by sutures and adhesive plaster.

The following day the pain seemed to be as bad as ever; and although pain experienced in any locality does not always entirely cease on section of the nerve, yet I much feared that the disease existed beyond the reach of the knife. However, the pain decreased every day, and the ulcerations on the leg began to heal immediately, and have now for some weeks disappeared. She has not suffered from any pain in the limb for more than eleven weeks. Her general health has in the mean time greatly improved; and her countenance, as might be inferred, has entirely altered its character.

I may mention a somewhat curious fact connected with this case—namely, that although the limb, before section of the nerve, was most sensitive to the prick of a needle, yet that a galvanic current of considerable strength, and applied in every conceivable direction, had not the slightest effect on it.

It is hoped that a mechanical contrivance now in the course of construction will enable this patient to dispense with crutches, upon the aid of which she has been for some years dependent.—(*London Lancet,* Oct. 1st, 1859.)

5. CASE OF SPINA BIFIDA TREATED BY COLLODION. By *Dr. Behrend.*—A strong healthy child was brought to Dr. Behrend when seven weeks old, having a swelling over the last lumbar vertebra and the upper part of the sacrum. It was the size of a small orange, of a roundish form with a broad base, and disappeared under pressure of the finger. The skin over it was very delicate, transparent, and of a palish red. Pressure which caused the disappearance of the fluid seemed to give the child pain, and induced distortion of the features, which disappeared when this was removed. The aperture in the vertebra could be distinctly felt. The author resolved to try the effect of compression by collodion; and in order that its action should not be too suddenly energetic, he mixed it with some castor-oil in the proportion of three parts to six of collodion. On July 2 the whole of the surface of the tumor and some distance beyond were painted with this. No bandage was applied, and the parts were left freely exposed to the air for an hour and a half, a firm but yet soft and yielding covering having formed over the tumor. Some wadding and sticking-plaster were now applied. Next day some amount of contraction was thought to have taken place, and a penciling with eight parts of collodion to two of castor-oil was performed. By the 7th of July a remarkable degree of contraction of the tumor had taken place, and it was painted with pure collodion. On the 8th there was a slight rupture observed in the shriveled wall of the tumor, and some moisture issued; but on close examination it was believed that the rupture only implicated the layer of the collodion, and did not penetrate within the tumor. This had now diminished to the size of a filbert, and became much flattened. A small plate of caoutchouc, wrapped in muslin, was laid over it and kept on by a roller. This was kept on for three weeks, when nothing else was visible than the thick skin, stretched tightly over the aperture in the spine, and from which the collodion had long since become separated. The plate was directed to be worn some time longer yet, and the child, when seen, (October 12,) seemed well and hearty, and fully developed—the large fontanelle, which had been too open, having become diminished in size. No trace of the tumor was visible, some thickened skin and a subcutaneous mass of almost cartilaginous hardness supplying its place, allowing the edges of the bony aperture only to be imperfectly felt. The author thinks that probably the collodion might have been used at once without the castor-oil, when its influence would have been more powerful; and he suggests, under certain circumstances, the combination of lead or tannin with it, in order to produce a more immediate effect on the wall of the tumor. In the present case he considers that some calomel, which, together with cold applications to the head, were employed on account of a head affection which threatened to come on during the treatment, may have contributed to the rapid absorption of the fluid.—(*Journal für Kinderkr.*, Band xxxi., p. 400; *Medical Times and Gazette*, July 2, 1859.)

6. ON FOREIGN BODIES IN THE URETHRA AND BLADDER. By *Professor Pitha.* (Wien Medizin. Wochenschr., 1858, Nos. 50, 51, 52.)—The immediate cause of this communication was an interesting case, in which, on account of the introduction of foreign bodies into the bladder, the operations of lithotomy and lithotrity were successively performed on the same individual. He was a soldier, aged thirty-four, who came into the hospital in consequence of a piece of lead-pencil, about three inches long, and pointed at either end, having slipped into the bladder while he was trying to pass it, as a substitute for a bougie. He had suffered excessive torment for a week, and the pencil (which was found divided into two parts) was removed by the lateral incision. He did very well. In two years he returned to the clinic, suffering from intense cystisis, brought on from the presence of a piece of sealing-wax which had entered the bladder during his manipulation with it. The patient protested against the repetition of lithotomy, which, indeed, would have hardly been advisable in the inflamed condition of the bladder, and it was resolved to have recourse to lithotripsy. He was brought under the influence of chloroform with great difficulty, and never to an extent sufficient to subdue the irritability of the bladder, which instantly rejected the smallest quantities of water which were thrown into it. The preliminary injection of the bladder had, therefore, to be dispensed with. The foreign body was seized with the greatest ease, and, not to pursue the details, was crushed and entirely removed in the course of three *séances*, at intervals of two or three days. The patient completely and rapidly recovered. This case is very interesting, from the fact of there being no means of anticipating the amount of resistance which wax that had lain in the bladder for four weeks would offer; and further, by showing that lithotripsy may be safely performed, notwithstanding violent general reaction and the greatest irritation of the bladder, producing complete intolerance of the presence of any water whatever. Hitherto one of the most received axioms has been, not to undertake the operation in an empty bladder. It may surprise some that the narcosis was not pushed to the extent of appeasing this irritability of the bladder, but repeated experience has shown that the highest doses of anæsthetic agents will not effect this.

The case is also interesting as exhibiting an example of the unexpected slipping of foreign bodies into the bladder having occurred twice in the same individual. This accident has not excited the attention its frequency and importance demands. If experience upon this point were collected, surprise would be excited at the extraordinary character of various objects found in the urethra and bladder,* of some of which it would be difficult to explain how they could be forced through the urethra, to say nothing of their slipping into the bladder. But even with respect to the commoner objects, most of which have some resemblance in shape or size to the catheter, such as pen-holders, pencils, glass tubes, metallic rods, pieces of wood, etc., one can scarcely conceive how, once introduced into the urethra, they should escape from the fingers and slip into the bladder. This may be intelligible enough as regards the straight, short, female urethra, but not so as to the male urethra, whose long curved canal is traversed with difficulty by a well-oiled catheter in inexpert hands. Some have sought for an explanation in a suction-action or a peristaltic movement of the urethra, but ample experience in introducing instruments does not favor this view. On the contrary, a powerful expelling influence is often exerted by the urethra, the instrument being forcibly ejected when it has reached the neck of the bladder, this resistance being in fact met with, more or less, in the widest urethræ. The walls of the canal are naturally closely applied to each other, and its entire mechanism is directed so as to favor the passage from within outward, and not the reverse of this. Demarquay has furnished this natural explanation, that the entrance of the body takes place at the period of erection, and especially of ejaculation. The urethra is then elongated, its walls are expanded and smooth, and its canal is gaping and well lubricated, while its curvatures are diminished. The penis in relaxing forces the body deeper inward, and the efforts of the person to prevent the occurrence by forcing back the penis, in order to shorten it, only add to the mischief. This seems the most rational explanation, for, in fact, these cases usually occur in onanists, who, in order to induce ejaculation, penetrate deeper and deeper into the urethra, having already blunted the sensibility of the anterior portion. In the performance of catheterism, too, we sometimes find, in spite of the greatest skill, the passage of the instrument is obstructed. Presently, the repeated attempts induce erection, and the instrument at once passes on. Sometimes the catheter glides at once to the membranous portion, where it meets with insurmountable resistance. Believing that there must be a fold or a stricture, or that a faulty direction has been observed, we seek to withdraw the instrument. This, however, is impossible without violence, so fast and immovably is it held; and now a phenomenon which astonishes the inexperienced is observed, viz.: the spontaneous deeper penetration of the instrument, as if propelled by some unseen power, a circumstance which has probably given rise to the suction theory.

---

* See a paper by M. Denucé, Brit. and For. Med.-Chir. Rev., vol. xx.

It is, however, a mere mechanical effect of the elastic return of the penis, (after having been forcibly drawn forward,) during which the muscular walls, closely embracing the instrument, are also carried backward. So strong may this spasmodic action of the muscles be, that nothing but rude force will overcome it, unless we wait for minutes or hours, until the spasm subsides. It may play the chief part where foreign bodies slip into the bladder during erection, at all events detaining them in the membranous portion of the urethra; and it will be the more certainly brought into action the more pointed and irritating the body be, especially as the state of erection increases the irritability of the urethra. It is well known that catheterism after coitus or pollution is much more difficult, and that in such case a seizure of the instrument most readily takes place, even in patients accustomed to the operation—a fact of some importance in the treatment of stricture.

For the removal of foreign bodies of a roundish shape from the urethra, such as beads, beans, calculi, etc., Professor Pitha has long been in the habit of using delicate long-bladed forceps, (*Kornzange*,) which can be easily manœuvred with one hand, while the fingers of the other fix the foreign body from behind. Several such forceps, of different lengths and widths, should be at hand, in order to choose from in particular cases. When the body is seated very deeply, as at the neck of the bladder, the instrument should have a gentle catheter-like curvature given to it; and such an instrument the author finds admirably adapted for the removal of fragments from the urethra after lithotrity. When the foreign bodies are too voluminous, they should be first broken up by means of Segalas' urethral *brise-pierre*, and when not capable of being broken, they must be removed by the button-hole operation, the wound in these cases always readily healing, even when situated in the perineum. In the case of sharp, pointed bodies, such as needles, awls and the like, special manipulation is required. When a needle is in the region of the penis, we must ascertain by touch its exact position, and then, by pressure on the blunt end, force the point through the walls of the urethra, until it can be seized by a forceps. Pins should be thrust through in the same manner, and so manœuvred that the head is directed toward the mouth of the urethra, and then removed by an urethral forceps. When the needle is implanted lower down in the urethra, we must act in the same manner with the fingers placed in the rectum. In bodies having yielding stems and blunt points, such as hair-pins, these procedures are impossible, and for the removal of such, an instrument contrived by Matthieu, of Paris, answers admirably. Leroy has modified the instrument, so that it now resembles the *brise-pierre à pignon*. Smooth, cylindrical bodies, such as needle-cases, pen-holders, bougies, etc., soon pass into the bladder. Before this they may usually be removed with ease, providing their progress backward be at once prevented by compression. Even if the body has reached the membranous portion of the urethra, it can be removed by pressing it forward through the rectum, while the curve of the urethra is diminished by traction of the penis. The cases wherein an instrument breaks in the urethra may be easily managed in this way with patience and presence of mind.

When the foreign bodies have reached the bladder, they are extracted with greater difficulty the longer and thinner they are. After they have remained in the bladder, too, they become incrusted, and are more difficult of removal, especially when the bladder has been rendered irritable. It is a matter of great difficulty to seize them in the longitudinal direction, but the extractor of Luer is admirably adapted for regulating the direction in which the body shall be removed, and with its aid our difficulties are reduced in these cases to the discovery of the foreign body.—(*British and Foreign Medico-Chirurgical Review*, No. 47, July, 1859.)

